# Clinical experience using a video‐guided spirometry system for deep inhalation breath‐hold radiotherapy of left‐sided breast cancer

**DOI:** 10.1120/jacmp.v16i2.5218

**Published:** 2015-03-08

**Authors:** Wensha Yang, Elizabeth M. McKenzie, Michele Burnison, Stephen Shiao, Amin Mirhadi, Behrooz Hakimian, Robert Reznik, Richard Tuli, Howard Sandler, Benedick A. Fraass

**Affiliations:** ^1^ Department of Radiation Oncology Cedars Sinai Medical Center Los Angeles CA USA

**Keywords:** breast, DIBH, respiratory motion, MV cine, EPID, cardiac dose

## Abstract

The purpose was to report clinical experience of a video‐guided spirometry system in applying deep inhalation breath‐hold (DIBH) radiotherapy for left‐sided breast cancer, and to study the systematic and random uncertainties, intra‐ and interfraction motion and impact on cardiac dose associated with DIBH. The data from 28 left‐sided breast cancer patients treated with spirometer‐guided DIBH radiation were studied. Dosimetric comparisons between free‐breathing (FB) and DIBH plans were performed. The distance between the heart and chest wall measured on the digitally reconstructed radiographs (DRR) and MV portal images, d_DRR_(DIBH) and d_port_(DIBH), respectively, was compared as a measure of DIBH setup uncertainty. The difference (Δd) between d_DRR_(DIBH) and d_port_(DIBH) was defined as the systematic uncertainty. The standard deviation of Δd for each patient was defined as the random uncertainty. MV cine images during radiation were acquired. Affine registrations of the cine images acquired during one fraction and multiple fractions were performed to study the intra‐ and interfraction motion of the chest wall. The median chest wall motion was used as the metric for intra‐ and interfraction analysis. Breast motions in superior–inferior (SI) direction and “AP” (defined on the DRR or MV portal image as the direction perpendicular to the SI direction) are reported. Systematic and random uncertainties of 3.8 mm and 2 mm, respectively, were found for this spirometer‐guided DIBH treatment. MV cine analysis showed that intrafraction chest wall motions during DIBH were 0.3 mm in “AP” and 0.6 mm in SI. The interfraction chest wall motions were 3.6 mm in “AP” and 3.4 mm in SI. Utilization of DIBH with this spirometry system led to a statistically significant reduction of cardiac dose relative to FB treatment. The DIBH using video‐guided spirometry provided reproducible cardiac sparing with minimal intra‐ and interfraction chest wall motion, and thus is a valuable adjunct to modern breast treatment techniques.

PACS number: 87.55.kh, 87.55.ne, 87.55.tg

## I. INTRODUCTION

Postsurgery radiation therapy has been shown to significantly reduce locoregional recurrence and improve survival for breast cancer patients compared with surgery alone.^(1)^ However, long‐term follow‐up from multiple trials has found that patients receiving whole‐breast radiotherapy also have an increased risk of cardiac disease.^2^, ^3^ Given the potential for radiation‐induced cardiac toxicity, it is recommended that the irradiated heart volume be minimized to the greatest possible degree without compromising the tumor coverage.^(4)^

Two types of spirometry‐based techniques, involving voluntary and involuntary DIBH, have been developed to reduce cardiac doses. The involuntary breath‐hold uses active breathing control to manage patients' breath‐hold with a significant reduction of heart dose.^(5)^ The voluntary breath‐hold technique, using a spirometer with video guidance (SDX, Muret, France),^(6)^ has also shown promising results in lung cancer; however, there are only limited data for left‐sided breast cancer. This study reports our initial clinical experience in applying this spirometry‐based system to deliver whole‐breast radiation to patients with left‐sided breast cancer during voluntary DIBH. We describe here the detailed clinical protocol, dosimetric comparisons, systematic and random uncertainties of the technique, and assessment of inter‐ and intra fraction breath‐hold motion based on the MV cine portal images collected during radiation.

## II. MATERIALS AND METHODS

### A. SDX breath‐hold system

This device uses a spirometer to measure in real time the patient's pulmonary volume. During the preparation phase, the patient breathes through the spirometer to establish a stable breathing baseline and is then asked to perform DIBH to determine the breath‐hold volume. Once the patient's comfortable DIBH volume is determined, imaging and/or radiation treatment will be performed at this defined volume. Video goggles are used to guide the process by providing visual cues to the spirometry pattern.

### B. Clinical workflow

#### B.1 Patient selection

All patients were 18 years or older with histologically proven left‐sided breast cancer and were screened for DIBH as described below.

#### B.2 CT simulation

All patients were setup on a breast board with left arm up, head turned to the right and chin extended. An initial free‐breathing (FB) multislice CT was obtained with 2.5 mm slice thickness. A straight line delineating the edge of the approximated tangent fields (selected to minimize in‐field lung volume) was then drawn on the CT, and slices involving heart were evaluated. If the line transected any portion of the cardiac silhouette, then the patient was selected for a second DIBH CT. The therapist first provided audio coaching to establish a stable ventilation pattern. Patients were then instructed to take a deep breath in and hold at a defined volume within a tolerance of ±0.1 L, and the time of sustained breath‐hold was defined according to individual patient's comfort. A minimal breath‐hold time of 20 s is required for the patient to be recommended for DIBH treatment.

#### B.3 Planning

The heart and left anterior descending artery (LAD) were segmented, following the Radiation Therapy Oncology Group heart atlas.^(7)^ Patients were treated with tangential fields, with or without a matching supraclavicular field. Typically a field‐in‐field technique was used to improve dose homogeneity, although, for some patients, multiple segments were allowed. While 6 MV was used for most patients, mixed energies (6 MV and 25 MV) were used as appropriate to reduce hot spots for some patients (typically the weighting of a 25 MV field was less than 5%). The AAA algorithm with heterogeneity correction within the Eclipse (Varian Medical Systems, Palo Alto, CA) planning system was used for final dose calculation.

#### B.4 Treatment

Twenty‐three patients were treated on a 23EX linear accelerator (Varian Medical Systems) commissioned with 6 MV and 25 MV X‐rays and an electronic portal imaging device (EPID). Five patients were treated on a Trilogy linear accelerator (Varian Medical Systems) commissioned with 6 MV and 16 MV X‐rays, cone‐beam computed tomography and EPID, though only EPID images were used in this study. An imaging‐only session was done on the first day. Patients were aligned to their CT‐simulation tattoos, and pretreatment MV portal images were taken while the patient was holding their breath at the predefined volume. For subsequent fractions, the same setup procedure was used with pretreatment portal images taken once a week, alternating between medial and lateral fields. Additionally MV cine images were taken during treatment delivery. After setup verification, patients were instructed first to breathe normally to establish a stable spirometry baseline, and then to take a breath into the predefined pulmonary volume. The radiation beam was then manually turned on by the therapist once the defined volume was reached. If the patient breathed out of the predefined tolerance (typically ±0.1 L), the radiation beam would be turned off manually by the therapist. The patient was then instructed to breathe normally to reestablish the spirometry baseline and then to breathe into the defined volume to complete the remaining treatment.

### C. Patient information

Twenty‐eight patients were treated with DIBH radiotherapy under an IRB‐approved protocol. Seven patients underwent mastectomies. Twenty‐one patients had breast conserving surgeries.

### D. Dosimetric analysis

The prescriptions ranged from 42.6 Gy to 50.4 Gy with 1.8–2.7 Gy/fraction. Tangential plans were designed on the DIBH CT. Rigid image registration between the DIBH and FB CTs was performed focusing on the breast. The DIBH plan was then copied to the FB CT and recalculated with the same MU for dosimetric comparisons. Dosimetric parameters, including mean dose for the heart and LAD, maximum dose for the LAD, and the percent volume receiving $\geq 20\;\text{Gy}(V_{20})$ for left lung, were analyzed. A two‐tailed paired *t*‐test was used with a p‐value of <0.05, defined as statistically significant.

### E. Portal imaging analysis

Weekly pretreatment portal images were taken, while daily MV cine images were performed for the first week of the treatment, or until a stable setup (defined as approved cine images for three consecutive days) was established. Portal images that were deemed unacceptable were retaken on the next day of the treatment. The frequency of the MV cine was reduced to twice per week for subsequent weeks. Generally each patient had four to five pretreatment MV ports (two to three from medial and two from lateral fields).

**Figure 1:**
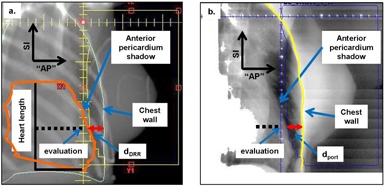
Schematic definition of $d_{\text{DRR}}$ (a) and $d_{\text{port}}$ (b).

The reproducibility of the heart displacement relative to the chest wall was evaluated by comparing the pretreatment DIBH portal images to the respective DRRs. The distance between the chest wall and the anterior pericardium shadow, defined in Fig. 1, was chosen as the metric for heart reproducibility during DIBH. These distances were named d_DRR_(DIBH) and d_port_(DIBH) for DRR and portal images. The difference between d_port_(DIBH) and d_DRR_(DIBH), $\Delta d=d_{port}(DIBH)-d_{DRR}(DIBH)$, was defined as the systematic uncertainty of the heart position in DIBH from simulation to treatment for a particular patient. The standard deviation of the Δd was defined as the random uncertainty. The mean and standard deviation values of Δd for the whole patient group are reported in both raw and absolute values to show the range of the uncertainties for the group, as well as to eliminate the effect of positive and negative values cancelling each other. “AP” is defined as the central–distal direction in the beam's eye view of the tangential fields. SI is defined as the conventional superior–inferior direction.

The displacement of the heart was also measured on the FB CT and defined as d_DRR_(FB). The difference between d_DRR_(DIBH) and d_DRR_(FB) was calculated to evaluate the relative heart shift in the “AP” direction. The shift in the SI direction was measured by comparing the CT coordinates of the most superior slices of the heart contours on FB CT and DIBH CT, since the scans were registered according to the location of the breast.

MV cine images were taken at time intervals representing 5% of the total MU during treatment (no additional patient dose was involved). The dose rate was set at 300∼500 MU/min, since there is a trade‐off between the number of images obtained during treating each field and the optimal time to ensure one field can be treated during one breath‐hold. For patients with an extensive number of segments, two breath holds per field were sometimes required. This results in a frame rate of 1 frame/5–10 s. The portal imaging system has a pixel resolution of 0.39–0.78 mm at the imager plane (depending on the treatment machine), which translates to 0.24–0.57 mm at the isocenter.

The MV cine images from 24 patients with complete datasets were selected for the intrafraction reproducibility analysis, based on the level of modulation resulting in a sufficient number of MV cine frames in one breath‐hold. In each MV image, the chest wall was automatically segmented using a MATLAB (MathWorks Inc, Natick, MA) program developed in‐house. Within each fraction, the movement associated with the chest wall was measured through an affine registration algorithm which compared the motion between the last frame and all the other frames in the same cine set. For each frame, the motion was assessed by transforming the pixels in the segmented chest wall via an affine matrix to match the chest wall in the last frame. The SI and “AP” shifts of each pixel were measured. The median SI and “AP” motions of the chest wall pixels were calculated for each available beam within each individual fraction and defined as intrafraction DIBH motion. The average number of frames analyzed per fraction was 6 (2–20).

Nineteen out of 28 patients were selected for the interfraction analysis. The selection of the MV cine images was based on the availability of at least 3 fractions of usable images and the level of modulation which results in a sufficient amount of chest wall that can be visualized in the image. The interfraction motion analysis uses the last frame of each fraction for a given patient and beam. The frame that has the closest chest wall spatial match to the DRR was used as the reference and compared to the last frame of the MV cine set from each fraction. The average number of fractions analyzed per patient in the interfraction analysis was 7 (3–15). Median SI and “AP” motion were obtained in the same way as the intrafraction analysis.

## III. RESULTS

All patients were able to complete the treatment course. Three patients had difficulties reaching a stable baseline during the first week of the treatment, but all were able to control breathing for the following weeks. Another patient had continued difficulty for two weeks and stayed on the couch for more than 30 min every day. It was decided to increase the breath‐hold tolerance to ±0.2 L in order to reduce the treatment time. For this patient, pretreatment ports were performed to verify the setup and MV cine was used on every fraction to monitor the breath‐hold reproducibility. All of the patients' DIBH related statistics are reported in Table 1. The mean DIBH volume for the patient group was 1.9 (0.9–2.6) L, with a standard deviation of 0.5 L. The mean achieved single breath‐hold length was 25.2 (16–34.6) s, with a standard deviation of 4.1 s. The mean relative heart shifts from FB to DIBH were 1.3 (0.1–2.5) cm and 3.2 (1.5–5) cm in “AP” and SI directions, respectively.

**Figure 2:**
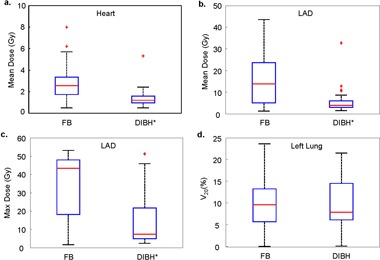
Comparisons of dosimetric parameters between FB and DIBH plans: (a) heart mean dose; (b) LAD mean dose; (c) LAD maximum dose; (d) left lung V_20_. Asterisk (*) indicates statistically significant.

As shown in Fig. 2, statistically significant dose reduction was observed for the heart and LAD when using the DIBH technique. On average, from FB to DIBH plans, heart mean doses decreased from 2.8 (0.5–8) Gy to 1.4 (0.5–5.3) Gy, LAD mean doses decreased from 16.2 (1.2–43.6) Gy to 5.8 (1.4–32.7) Gy, and LAD maximum doses decreased from 35.4 (1.6–53) Gy to 15.8 (2.3–51.2) Gy. A slight (0.4%) reduction in left lung $V_{20}$ was observed, but it was not statistically significant.

**Figure 3:**
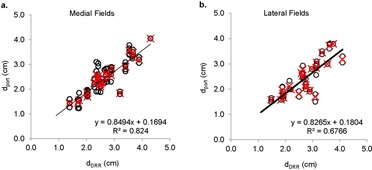
Scatter plots of $d_{\text{port}}$ vs. $d_{\text{DRR}}$, calculated from medial (a) and lateral (b) fields. Open circle (O) represents a comparison from one fraction. Red crosses (x) represent the average of all fractions for an individual patient.

Differences between $d_{\text{DRR}}$ and $d_{\text{port}}$ are shown in Fig. 3 for both medial and lateral fields for all patients. Each open circle represents a comparison between $d_{\text{port}}$ for one fraction and its respective d_DRR_. The red crosses represent the average $d_{\text{port}}$ from different fractions for each patient. A line fit was created from the average $d_{\text{port}}$ vs. d_DRR_. The slope of the linear regression describes how much the $d_{\text{port}}$ and $d_{\text{DRR}}$ are in agreement (i.e., slope of 1 indicates that they agree with each other all the time, and slope <1 indicates that $d_{\text{DRR}}>d_{\text{port}}$). Slopes of 0.85 and 0.83 were observed for the comparisons on medial fields and lateral fields, respectively. Patients' DIBH volumes and their systematic and random uncertainties are reported in Table 2. Across all patients, the mean systematic uncertainty calculated from the raw Δd is −0.26(−1.7±0.75) cm, with a standard deviation of 0.42 cm. The mean systematic uncertainty calculated from the absolute Δd is 0.38(0±1.7) cm, with a standard deviation of 0.32 cm. The mean random uncertainty of Δd for each patient is 0.21 (0.08–0.48) cm, with a standard deviation of 0.11 cm.

**Table 1:** DIBH volume, achieved single breath‐hold lengths of breath‐hold during treatment and relative heart shifts from FB to DIBH in “AP” and SI directions

*Measurements*	*DIBH Volume (L)*	*Single Breath‐hold Lengths (s)*	*Heart Shift in “AP” (cm)*	*Heart Shift in SI (cm)*
v±σ	1.9±0.5	25.2±4.1	1.3±0.7	3.2±0.8
(range)	(0.9–2.6)	(16.0–34.6)	(0.1–2.5)	(1.5–5.0)

Average of the patient cohort; σ=standard deviation of the patient cohort; FB=free breathing; DIBH=deep inhalation breath hold; SI=superior‐inferior; “AP”=the direction perpendicular to the SI direction on the DRR and MV portal images.

**Figure 4:**
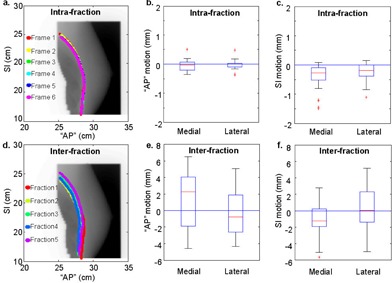
Overlays of chest wall traces for a typical patient and the box plots of intrafraction ((a) to (c)) and interfraction ((d) to (f)) chest wall motion for the patient cohort, calculated from medial and lateral fields. Submillimeter intrafraction motion and small (−5) interfraction motion are depicted by the overlaps of the chest wall traces derived from the multiple frames in one breath‐hold and in different breath‐holds from multiple days for this example patient.

Figure 4 shows the intrafraction ((a) to (c)) and interfraction ((d) to (f)) motion analysis (positive values signify “anterior” or inferior motion). Most patients present parallel intra‐ and interfraction chest wall patterns, as shown in Figs. 4(a) and 4(d). The mean patients' intrafraction median “AP” chest wall motion is −0.04(−0.4±0.5) mm for those calculated from medial fields and −0.02(−0.4±0.5) mm from lateral fields. The mean patients' intrafraction median SI chest wall motion is −0.4(−1.5±0.1) mm for medial fields and −‐0.3(−‐1.1±0.2) mm for lateral fields. Overall absolute intrafraction motion combining both medial and lateral fields is 0.3(σ=0.4) mm in “AP” direction and 0.6(σ=0.7) mm in SI direction, which are close to the portal imager resolution. The mean patients' interfraction median “AP” chest wall motion is 1.3(−4.6±6.5) mm for those calculated from medial fields and −0.3(−4.3±5.1) mm for those calculated from lateral fields. The mean patients' interfraction median SI chest wall motion is −1.2(−5.7±2.8) mm for medial fields and 0.2(−4.9±5.2) mm for lateral fields. Overall absolute interfraction motion combining medial and lateral fields is 3.6(σ=2.7) mm in the “AP” direction and 3.4(σ=2.8) mm in the SI direction.

**Figure 5:**
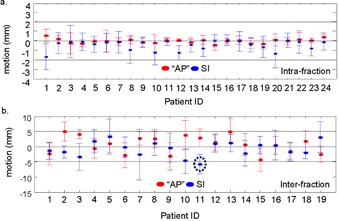
Mean and standard deviations of intrafraction (a) and interfraction (b) motions calculated from all image registrations for each individual patient.

Figure 5 shows individual patients mean and standard deviations of intra‐ and interfraction motion in “AP” and SI directions, calculated from all image registrations for each patient. All patients stayed in the −2 to −2 mm range for intrafraction motion. All but one patient (denoted by a dotted circle) stayed in the −2 to 5 mm range for interfraction motion.

**Table 2:** Statistics of $d_{\text{DRR}}$ (DIBH) and $d_{\text{port}}$ (DIBH)

*Measurements*	*Systematic Uncertainties (cm) From Raw* Δd				*Systematic Uncertainties (cm) From Absolute* Δd				*Random Uncertainties (cm)*			
	*Mean*	*Max*	*Min*	$\sigma_{s}$	*Mean*	*Max*	*Min*	$\sigma_{s}$	*Mean*	*Max*	*Min*	$\sigma_{r}$
All patients	−0.26	0.75	−1.70	0.42	0.38	1.70	0	0.32	0.21	0.48	0.08	0.11

σ=standard deviation; $\sigma_{s}=\text{standard deviation of the systematic uncertainties}$; $\sigma_{r}=\text{standard deviation of the random uncertainties}$.

## IV. DISCUSSION

Many techniques have been proposed for DIBH radiation of left‐side breast cancer to minimize cardiac doses, including involuntary breath‐hold with active breathing control,^(8)^ and voluntary breath‐hold using various devices.^9^, ^10^, ^11^, ^12^, ^13^ McIntosh et al.^(13)^ reported voluntary deep inhalation breath‐hold using an RPM signal as a surrogate, in which 43%–60% dose reductions of cardiac doses were observed. Nissan and Appelt^(14)^ reported a statistically significant mean heart dose reduction of 48%, and a small reduction of lung $V_{20}$ with marginally significant p‐value (<0.04). Our observation of cardiac dose reduction is similar, but slightly larger, than these reported observations, ranging from 50% to 64%. Similar to the Nissan report, we also observed a small reduction of lung $V_{20}$, although statistically insignificant. The slight difference in our dosimetry data compared to those reported in the literature can be a result from different oncologists and planners defining the tangential fields and contours in the initial treatment planning, which can lead to different baseline dosimetry.

McIntosh et al.^(13)^ used displacement difference from spinal cord to sternum across the isocenter between coregistered planning DRR and kV imaging as a measure of breath‐hold reproducibility, and the difference between bony registration and heart registration was used as a measure of heart shift. These are valid measures to analyze interfraction motion; however, they cannot provide residual intrafraction motion during beam‐on. Jagsi et al.^(15)^ reported displacements (3 mm in AP and 6 mm in SI) of LAD position under shallow breathing states using active breathing control and the images were acquired at the time of the CT simulation. Moran et al.^(5)^ also used CT‐simulation images to study the short‐term displacement and reproducibility of the breast or chest wall and nodal regions at different breath‐hold states using ABC, in which −4 of reproducibility was observed. Although using CT images to analyze residual breast motion under breath‐hold states is a useful method, the relative low slice resolution (typically 2.5 mm in SI direction) might limit this method from detecting motion that is smaller than the slice resolution. Our study uses high‐resolution (submillimeter) MV cine images directly obtained from the treatment during beam‐on, which can better represent the true treatment situations. Our data show only submillimeter displacements in the chest wall under the DIBH state using this video‐guided spirometry system, which indicates that the uncertainties induced by patients' breathing can be further improved with breath‐holds and quantifies the improvements that one could expect. One might not be able to make such conclusions if CT images with relatively low slice resolution were used. To our knowledge, this is the first study using MV cine images to evaluate intra‐ and interfraction motions of spirometer‐based DIBH treatment for left‐sided breast patients. Another novelty in our study is the use of affine registration on the analysis of MV cine images, which allowed the analysis to be performed on a large chest wall region with an average of 154 pixels (landmarks for the entire chest wall length along the image) in each image for analysis, while most other studies focused only on one or a handful of landmarks in the images. The affine algorithm takes chest wall deformation into consideration, which is also a new result that has not been reported elsewhere.

In our study, the setup accuracy analysis from the pretreatment images shows that many patients had a systematic deviation from simulation to treatment. This might be a result of the following:

1. Heart position is taken into consideration when designing the tangent fields to maximize $d_{\text{DRR}}$ without compromising the tumor dose coverage; however, patient rotation during DIBH at the time of the treatment can lead to a consistently smaller $d_{\text{port}}$ compared to d_DRR_;
2. Imaging qualities, such as resolution and signal‐to‐noise ratio, are different between the DRR and MV ports;
3. Identification of the heart is different on the two imaging modalities, with one from the CT contour projected to DRR and the other from the pericardium shadow on the MV portal images.

Based on these differences, a mean 0.38 cm systematic uncertainty is reasonable. One patient in the cohort was significantly different (Δd=1.7 cm) from the rest of the group. The mean systematic uncertainty over all patients is reduced to 0.32 cm when this patient is removed from the analysis. Although the mean random uncertainty is only 0.21 cm for this patient group as a whole, some patients can have as large as 0.48 cm random uncertainty between fractions. It is recommended to monitor patients' breathing by performing MV cine imaging more frequently for these patients, since no additional dose is used in the cine imaging.

Most patients showed parallel chest wall patterns from different days. As shown in Fig. 4(d), it should also be noted that some patients' chest walls may exhibit different shapes during DIBH in different fractions. This might also be a result of patient rotation. All of the patients reported in this study were lying supine on the slant board with no restraint. However, it has been reported that with use of a Vac‐Lok bag (Elekta Medical Intelligence, Atlanta, GA), the correlation coefficient between $d_{\text{DRR}}$ and $d_{\text{port}}$ is higher than for those without a Vac‐Lok bag, which leads to a reduced setup uncertainty.^(16)^ Other studies also have reported that the use of cradles for breast patients can improve the reproducibility of the setup.^(17)^

Although the mean intrafraction median chest wall motion for the current patient group is small (−0.2±0.8 mm), the range of median chest wall motion across all intrafraction comparisons was ‐8.9 mm to 3 mm. The largest intrafraction motion (‐8.9 mm) is an outlier from one fraction with most of the values clustering between ‐2 to 2 mm. It is worth noting that patient intrafraction motion can vary significantly on some image frames taken during beam‐on. Close examination of the outliers, which we defined as less than the 2.5% quartile or greater than the 97.5% quartile, shows that of these outliers 27/60 intrafraction comparisons in the “AP” direction and 41/60 in the SI direction are comparing the first frame to the last frame. This suggests that the manual‐controlled beam‐on process was not optimal, and that the radiation beam was turned on too soon for some fractions. Similarly for all interfraction comparisons, a small mean (0.1±4.4 mm) motion was observed with a large range (−16.7±12.8 mm). Daily tattoo‐based setup uncertainties without pretreatment imaging setup corrections might contribute to the large range of the interfraction motion.

**Figure 6:**
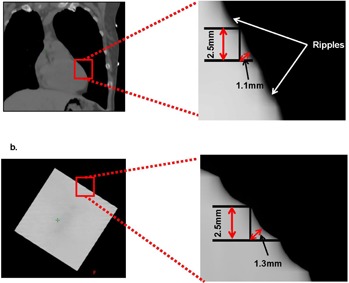
Ripple patterns of a patient's heart (a) and a static phantom (b) in CT images.

DIBH provides a fairly reproducible relative displacement of the heart; however, the effect of involuntary cardiac motion is unknown. In an attempt to estimate the cardiac motion, we reviewed the heart in the coronal plane on the DIBH CT scans for all patients. As shown in Fig. 6(a), a ripple pattern with a distance of ∼2.5 mm in the SI direction between the ripples was observed, which agrees well with the CT slice thickness. A similar ripple pattern was observed on a static phantom scan with a slanted edge, as shown in Fig. 6(b). The blurring effect likely results from a combination of cardiac motion and noise in the CT reconstruction. The magnitude of this blurring for both the patient group and for the phantom is 1∼1.5 mm, which is significantly smaller than the mean relative heart shift provided by the DIBH. Therefore, the cardiac motion may not be a concern in these treatments.

For patients with supraclavicular fields, field matching can be challenging. Subtle differences in DIBH volume may lead to over‐ or underdosage at the match line with the tangential fields. Hence, providing therapists with the field outlines on the body surface is an important step in our protocol. Patients were instructed to breathe in to obtain the defined DIBH volume, and a second check of the field outlines on the skin was performed by the physician on the first day. On subsequent treatment sessions, the skin outlines were checked by therapists.

## V. CONCLUSIONS

Deep inhalation breath hold treatment of left‐sided breast cancer patients was successfully performed with a video‐guided voluntary spirometry system. All patients studied successfully completed their treatment courses using DIBH treatment. Evaluation of the DIBH plan compared to the free‐breathing plan shows statistically significant decreases in heart and LAD dose metrics over all patients.

Detailed analysis of the DIBH reproducibility showed an average of 0.38 cm systematic uncertainty and 0.21 cm random uncertainty. Intrafraction motions of 0.3 and 0.6 mm and interfraction motions of 3.4 and 3.6 mm were observed in “AP” and SI directions, respectively.

This system appears to provide reproducible cardiac sparing and reasonable intra‐ and interfraction chest wall motion and is a valuable adjunct to modern breast treatment techniques.

## ACKNOWLEDGMENTS

We thank Dr. Yong Yue for valuable discussion.
